# On-Demand Generation of a Formaldehyde-in-Air Standard

**DOI:** 10.6028/jres.102.037

**Published:** 1997

**Authors:** P. M. Chu, W. J. Thorn, R. L. Sams, F. R. Guenther

**Affiliations:** National Institute of Standards and Technology, Gaithersburg, MD 20899-0001

**Keywords:** catalytic conversion of methanol, formaldehyde, formaldehyde standard, gas standards, vehicle emissions

## Abstract

The feasibility of using catalytic conversion of methanol to formaldehyde to produce standard amount of substance fractions of formaldehyde was examined. The conversion efficiencies of several catalysts were measured as a function of temperature, balance gas, catalyst bed length, and methanol amount of substance fraction in an effort to identify conditions which yield high and consistent conversion of methanol to formaldehyde. The highest observed conversion rate was (97 ± 4) % using a molybdenum catalyst, where the error is the 2*σ* uncertainty. The conversion efficiency was found to be consistent over repeated cycles and over a long lifetime test, suggesting that a molybdenum catalyst is a viable candidate for a standard formaldehyde generator, particularly for low formaldehyde amount of substance fractions (< 15 μmol/mol).

## 1. Introduction

Formaldehyde, a chemical commonly found in many industrial and commercial environments, has been identified as a source of free radicals which lead to the formation of ozone and photochemical smog [1]. Emissions from gasoline-powered motor vehicles are a major source of ambient levels of formaldehyde [2]. To improve air quality, legislation has been enacted to reduce vehicle emissions by using alternative fuels and modifying the engines [3]. The efforts to correlate levels of formaldehyde and other vehicle emissions with various fuel mixtures rely on the preparation of accurate and precise standard amount of substance fractions to calibrate the analytical instrumentation. In fact, the American Industry/Government Emissions Research (AIGER) [4] consortium identified a practical formaldehyde standard as a critical technology needed to improve measurements of emissions from motor vehicles. This need for standard gas mixtures will further increase as emissions are reduced and measurements of lower amount of substance fractions are required.

A variety of methods have been used to generate calibrated mixtures of formaldehyde including permeation devices, diffusion cells, reaction vessels and static gas cells [5]. Unfortunately, these techniques are difficult to use in field environments because they require constant calibration. A pressurized cylinder which consistently delivers a known amount of substance fraction of formaldehyde would be an ideal standard for an industrial setting. However, formaldehyde vapor’s high reactivity and tendency to polymerize has limited the success of delivering formaldehyde gas standards in a cylinder [6]. As an alternative method, the feasibility of a cylinder-deliverable formaldehyde standard by catalytically converting methanol from cylinder standards was examined. If the methanol to formaldehyde conversion rate is well characterized, this method has the potential of delivering known amount of substance fractions of formaldehyde based on the initial amount of substance fraction of the methanol standard.

Passing a mixture of methanol in air over a heated catalyst has been a key process for manufacturing formaldehyde since the late 1800s [7]. Formaldehyde monomer was first synthesized and isolated by passing methanol vapors and air across hot platinum wire. Subsequently, copper and silver catalysts were used in large scale formaldehyde manufacturing. Additional catalysts such as iron, chromium, and molybdenum also react with methanol to give high formaldehyde yields [8, 9, 10]. Formaldehyde can be generated by oxidation,

CH3OH+12O2→CH2O+H2O (1)

and/or dehydrogenation of methanol,

$${\text{CH}}_{3}\text{OH}\to {\text{CH}}_{2}O+H_{2}.$$ (2)

Reactions which compete with the final formaldehyde yield include pyrolytic decomposition of formaldehyde to carbon monoxide and hydrogen, particularly at temperatures above 300 °C, and oxidation of formaldehyde to formic acid or to carbon dioxide and water.

In this study the methanol to formaldehyde conversion efficiency of several catalysts was measured using a tunable diode laser absorption spectrometer (TDLAS) and a Fourier-transform infrared spectrometer (FTS). The conversion efficiencies were examined as a function of catalyst temperature, balance gas, catalyst bed length and methanol amount of substance fraction in an effort to identify conditions which yield high and consistent conversion of methanol to formaldehyde.

## 2. Experimental

**Fig. 1:**
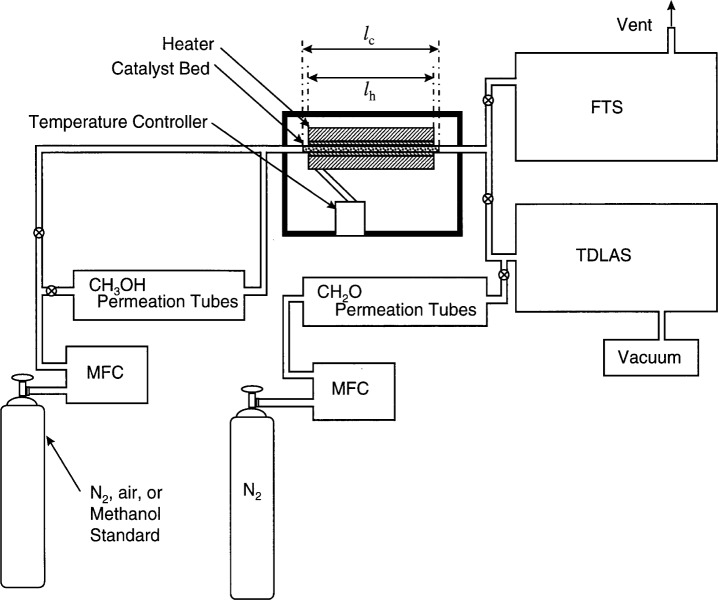
Schematic diagram of the formaldehyde generator, the methanol delivery, the tunable diode laser absorption spectrometer (TDLAS), and the infrared Fourier-transform spectrometer (FTS). The gas flow rates were controlled by mass flow controllers (MFC). The catalyst bed and heater lengths are denoted by *l*_c_ and *l*_h_, respectively.

The formaldehyde generator consisted of a 0.95 cm outer diameter stainless steel tube packed with one of the catalysts listed, along with the mesh sizes and the catalyst source, in Table 1. Ultra-thin high performance ceramic heaters [11]^1^ with a 3.18 cm inner diameter were used with a microprocessor temperature controller [2] to heat the stainless steel tube and the catalyst. A brass rod was machined to provide thermal contact between the ceramic heater and the stainless steel tube. A schematic diagram of the formaldehyde generator with the methanol delivery system, the TDLAS and the FTS is provided in Fig. 1.

In this study, two 20 cm long methanol permeation tubes [13] maintained at 50.0 °C and weighed periodically were used as one quantitative source of methanol. The permeation rates were obtained by modeling the permeation tube weight loss as a function of time by linear regression. The total methanol permeation rate was (6.96 ± 0.06) × 10^−6^ g/min. Unless otherwise noted, the uncertainties in this paper are expressed as an expanded uncertainty *U* = *ku*_c_ with *u*_c_ being estimated from the experimental standard deviations and the coverage factor *k* set equal to 2 [14]. A calibrated mass flow controller (MFC) was set to deliver the carrier gas, either ultra-high-purity (UHP) air or UHP nitrogen, at (1.00 ± 0.01) L/min across the permeation tubes. The final methanol amount of substance fraction delivered to the formaldehyde generator was (4.86 ± 0.06) μmol/mol. Gravimetric cylinder standards containing (4.54, 18.81, 88.8 and 202.2) μmol/mol methanol-in-air were used as a second quantitative methanol source. In this case, the relative expanded uncertainty in the methanol amount of substance fraction was 0.5 %. Delivering methanol from the cylinder standards under the continuous flow conditions minimized any possible adsorption effects.

Typical measurements consisted of continuously flowing a methanol gas mixture, slightly above ambient pressure, over the catalyst bed for 10 min to flush the system. The catalyst bed was then heated to the appropriate temperature and allowed to equilibrate for 15 min before any measurements were made. A 10 min equilibration time was used between each point in the temperature dependence studies. The amount of formaldehyde produced was measured using a lead salt tunable diode laser system which has been described in detail previously [15]. Briefly, approximately one-third of the gas exiting the formaldehyde generator was drawn into a 30 L variable length multipass sample cell. The cell pressure was maintained at approximately 1.33 kPa with a needle valve. The final pressure was monitored with a calibrated capacitance manometer determined to have a relative expanded uncertainty of 0.1 %. The cell path length was set to (69.53 ± 0.01) m. The lead salt tunable diode laser was tuned to a formaldehyde line in the 2920 cm^−1^ to 3000 cm^−1^ region. The diode was frequency modulated at 36.3 kHz and the laser intensity after the cell was monitored with a InSb detector. The signal was processed using a high speed lock-in amplifier in the second derivative mode.

The signal from the formaldehyde generator was compared to the signal obtained from one 10 cm and two 20 cm long formaldehyde permeation tubes [13] held at 90.0 °C. The formaldehyde permeation tubes were calibrated in the same manner as the methanol permeation tubes. The total formaldehyde permeation rate was (8.2 ± 0.2) × 10^−6^ g/min. A calibrated MFC was used to flow (1.00 ± 0.01) L/min of nitrogen across the formaldehyde permeation tubes producing a final formaldehyde amount of substance fraction of (6.1 ± 0.2) μmol/mol.

The amount of substance fractions of methanol and CO_2_ contained in both the initial gravimetric methanol samples and in the formaldehyde generator exhaust were measured using an FTS equipped with a multipass cell. The sample gas flowed through the room temperature cell continuously at 1 L/min and was maintained slightly above ambient pressure. Spectra were averaged over 30 min at 0.120 cm^−1^ nominal resolution using a HgCdTe detector with the cell path length set at (9.28 ± 0.01) m. Background spectra were acquired by flowing UHP nitrogen through the room temperature cell. The inteferograms were processed using a 3-term Blackman-Harris apodization function, Mertz phase correction, a factor of two zero-filling and a non-linear detector correction routine supplied with the instrument [16]. The final absorbance spectra were compared to corresponding quantitative spectra in the NIST spectral database [17] and the 0.5 cm^−1^ resolution quantitative spectra in the QASoft-96 database [18]. A computer program which distributed the intensity of each point in the spectrum over a gaussian of appropriate linewidth [19] was used to match the resolution of the spectra acquired at 0.120 cm^−1^ to the 0.5 cm^−1^ reference spectra.

## 3. Results and Discussion

**Fig. 2:**
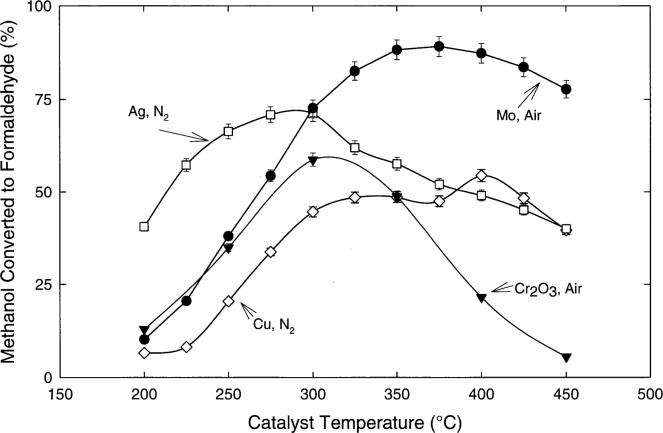
The percent methanol converted to formaldehyde measured as a function of temperature for a number of catalysts with *l*_c_ = 11.4 cm and *l*_h_ = 7.6 cm. The methanol amount of substance fraction was (4.86 ± 0.06) μmol/mol from methanol permeation tubes.

Figure 2 shows the percent methanol converted to formaldehyde as a function of temperature for copper, silver, chromium oxide, and molybdenum catalysts with the balance gas, UHP air or UHP nitrogen. These experiments used a catalyst bed length *l*_c_ = 11.4 cm, with a heater of length *l*_h_ = 7.6 cm, around the middle portion of the catalyst bed. The initial methanol amount of substance fraction was (4.86 ± 0.06) μmol/mol generated by permeation tubes. No formaldehyde was observed for nickel and iron oxide catalysts with either air or nitrogen as the carrier gas. For the copper and silver catalysts, formaldehyde was observed only when nitrogen was used as the carrier gas. Chromium oxide required air to produce formaldehyde, while molybdenum generated formaldehyde with either air or nitrogen as the carrier gas. From Fig. 2 it is clear that the formaldehyde production is strongly dependent on the catalyst, the catalyst temperature and the carrier gas.

Under the tested conditions, the molybdenum catalyst clearly gives the highest conversion of methanol to formaldehyde. Furthermore, in contrast to the copper catalyst, the performance of the molybdenum catalyst is not likely to degrade if it is exposed to air at high temperatures. Since only the formaldehyde product was measured, it is impossible to identify the underlying reasons for the relative methanol conversion efficiencies observed for the other catalysts tested. Possible reasons for the lower formaldehyde yields may include: 1) a lower methanol reaction probability; 2) methanol reacts to form other products, such as CO and H_2_; 3) formaldehyde further reacts to form other products. Previous work suggested that formaldehyde product is less likely from metals with high CO binding energies [20].

Although the surface structure of the metal granules was not characterized, insight into the reaction mechanism can be gained from ultra-high vacuum studies of methanol adsorption and reaction on a variety of single crystal surfaces. High resolution electron energy loss measurements reveal that a methoxy intermediate resides on several metal surfaces, including molybdenum [20, 21, 22]. Temperature programmed desorption studies indicate that methoxy intermediate decomposes by a C–H bond breaking followed by further decomposition to CO and H_2_ or formaldehyde desorption [23], depending on the surface. Residual carbon and oxygen can also remain on the surface.

**Fig. 3:**
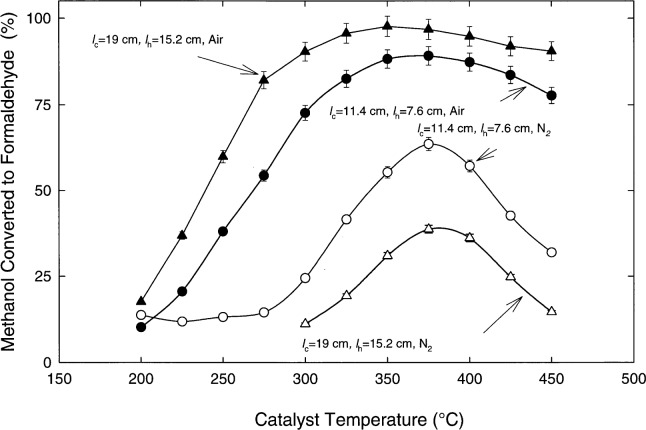
The percent methanol converted to formaldehyde as a function of molybdenum catalyst temperature using *l*_c_ = 11.4 cm with *l*_h_ = 7.6 cm and *l*_c_ = 19 cm with *l*_h_ = 15.2 cm. The methanol amount of substance fraction was (4.86 ± 0.06) μmol/mol from methanol permeation tubes.

Figure 3 shows the molybdenum catalyst conversion efficiency as a function of the balance gas. For a catalyst bed with *l*_c_ = 11.4 cm and *l*_h_ = 7.6 cm, the percent methanol converted to formaldehyde increases by 40 % using air rather than nitrogen as the balance gas. This clearly indicates that oxygen in the carrier gas plays an important role in the formation of formaldehyde for the molybdenum catalyst. The mechanism for this increased efficiency has not been identified. The presence of adsorbed oxygen may enhance the methoxy radical formation and/or subsequent formaldehyde generation. The oxygen may also provide an alternative oxidation reaction to form formaldehyde.

Figure 3 shows the increase in the methanol to formaldehyde conversion efficiency observed for a catalyst bed with *l*_c_ = 19 cm and *l*_h_ = 15.2 cm versus *l*_c_ = 11.4 cm and *l*_h_ = 7.6 cm. This indicates that a longer catalyst interaction time enhances the formaldehyde yield. A conversion efficiency as high as (97 ± 4) % was measured for a (4.86 ± 0.06) μmol/mol methanol standard with *l*_c_ = 19 cm, *l*_h_ = 15.2 cm, and the catalyst temperature held at 350 °C. The precision of this measurement is limited by the precision of the formaldehyde permeation tubes used to calibrate the TDLAS and the inherent instabilities of the particular diode used. The catalyst tube length was not lengthened further, since it is unlikely that a significant increase in the conversion efficiency for the (4.86 ± 0.06) μmol/mol methanol standard would be gained. It is interesting to note that the formaldehyde yield decreased for the longer tube with nitrogen as the carrier gas. In this case, the formaldehyde decomposes with the longer interaction with the catalyst in the nitrogen environment.

**Fig. 4:**
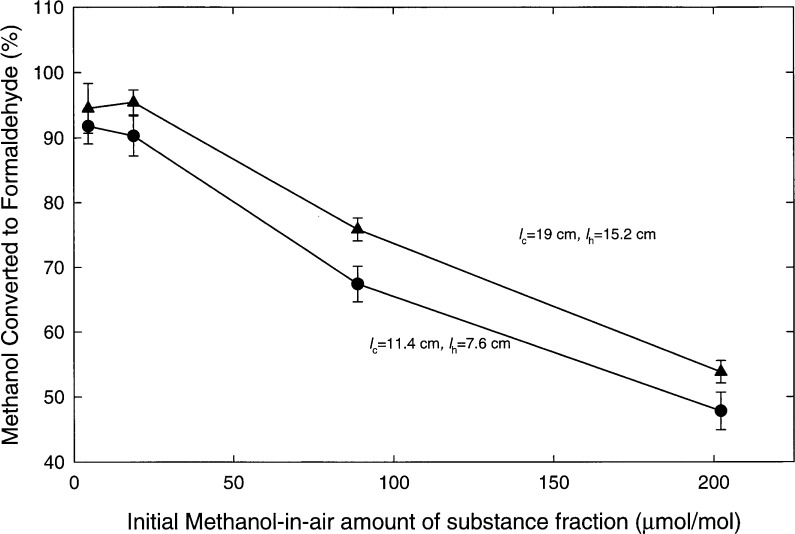
The percent methanol converted to formaldehyde measured as a function of the initial amount of substance fraction of methanol-in-air standards using a molybdenum catalyst with *l*_c_ = 19 cm and *l*_h_ = 15.2 cm.

The molybdenum catalyst conversion efficiency was examined as a function of initial methanol amount of substance fraction using four gravimetric methanol-in-air standards. As shown in Fig. 4, the percent methanol converted to formaldehyde decreased as the initial methanol amount of substance fraction increased from 5 μmol/mol to 200 μmol/mol. Again, the longer catalyst bed, with *l*_c_ = 19 cm and *l*_h_ = 15.2 cm, produced more formaldehyde than the shorter bed. This suggests that *l*_c_ and *l*_h_ can be further optimized to yield higher conversion efficiencies for higher initial methanol amount of substance fractions. Adjustment of the amount of oxygen in the carrier gas may also enhance the final formaldehyde yield. Additional experiments suggested that molybdenum catalyst that had been exposed to an oxygen environment at high temperatures over a long period gives higher conversion efficiencies at higher initial methanol amount of substance fractions.

**Fig. 5:**
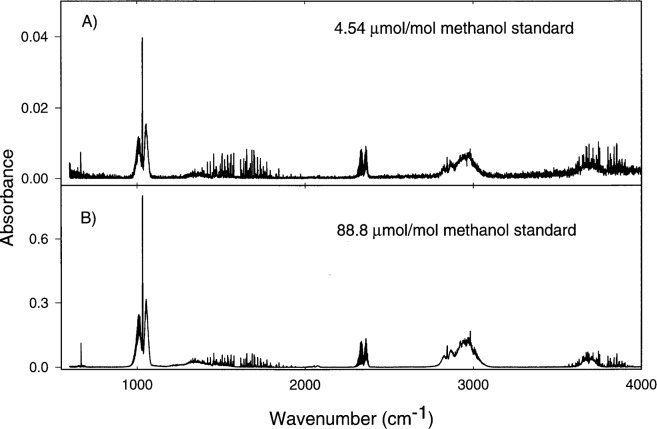
Fourier-transform infrared spectra of the initial methanol gravimetric samples. A) (4.54 ± 0.02) μmol/mol methanol-in-air gravimetric standard. B) (88.8 ± 0.1) μmol/mol methanol-in-air gravimetric standard.

**Fig. 6:**
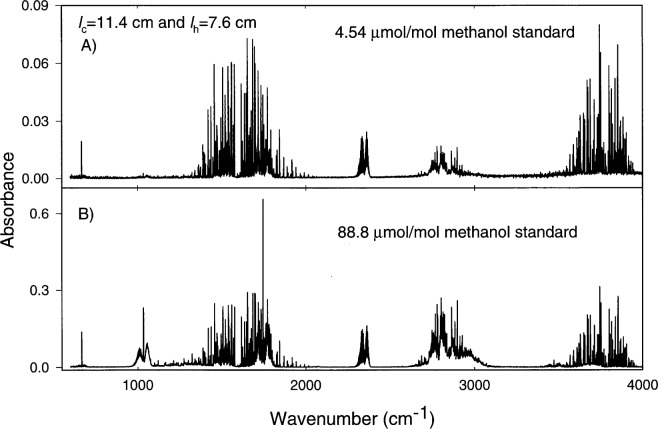
Fourier-transform infrared spectra of the outflow from a molybdenum catalyst bed with *l*_c_ = 11.4 cm and *l*_h_ = 7.6 cm. The catalyst bed was maintained at 350 °C with the methanol mixture flowing at 1 L/min. A) (4.54 ± 0.02) μmol/mol methanol-in-air gravimetric standard. B) (88.8 ± 0.1) μmol/mol methanol-in-air gravimetric standard.

**Fig. 7:**
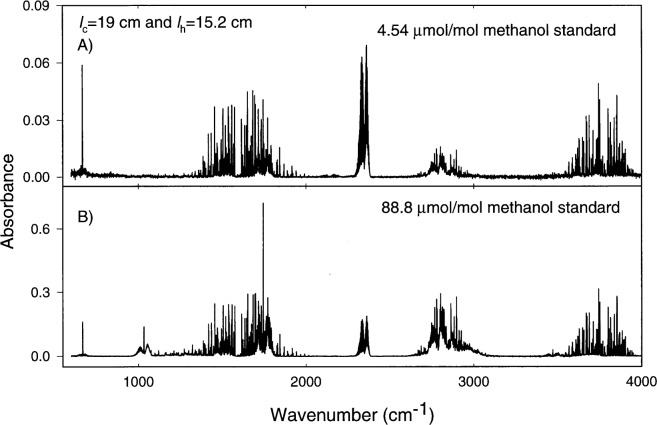
Fourier-transform infrared spectra of the exhaust from a molybdenum catalyst bed with *l*_c_ = 19 cm and *l*_h_ = 15.2 cm. The catalyst bed was maintained at 350 °C with the methanol mixture flowing at 1 L/min. A) (4.54 ± 0.02) μmol/mol methanol-in-air gravimetric standard. B) (88.8 ± 0.1) μmol/mol methanol-in-air gravimetric standard.

An FTS was used to identify additional products of the formaldehyde generator. Details of these measurements can be found in the Appendix. Figures 5, 6 and 7 show infrared spectra for the 4.54 μmol/mol and 88.8 μmol/mol methanol in air gravimetric standards and spectra of the formaldehyde generator exhaust for two molybdenum catalyst bed lengths. In all cases the catalyst was maintained at 350 °C with the methanol standard flowing at 1 L/min. The essential features of these spectra match the reference spectra [16, 17] of methanol, formaldehyde, and carbon dioxide. In all cases, less than 0.2 μmol/mol of carbon monoxide was detected. Comparisons of Figs. 6 and 7 with available reference spectra indicate that other potential byproducts such as acetaldehyde, formic acid, and methane, if present, are below the detection limits. At 0.120 cm^−1^ resolution, the bands of potential hydrocarbon byproducts overlap with the formaldehyde bands. Therefore, it is difficult to quantitate trace levels of these species in the presence of formaldehyde, particularly at higher final formaldehyde amount of substance fractions. Table 2 lists the band regions and the approximate FTS detection limits for acetaldehyde, formic acid and methane in the presence of formaldehyde at two different initial methanol amount of substance fractions. Clearly, these measurements would be improved by using a technique, such as higher resolution infrared spectroscopy, that can selectively detect a variety of small hydrocarbons.

Table 3 summarizes the amount of substance fraction of methanol contained in the initial methanol gravimetric standard and the final amount of substance fractions emitted by the formaldehyde generator based on the infrared spectra. For the (88.8 ± 0.1) μmol/mol methanol-in-air standard, a substantial amount of methanol remains after the converter. This supports the notion that the catalyst bed length could be further optimized to improve the conversion efficiency for higher initial methanol amount of substance fractions. To check whether the products in the formaldehyde generator output account for all of the methanol introduced into the system, the sum of the final methanol and formaldehyde amount of substance fractions is compared with the initial methanol amount of substance fraction in Table 3. Of course, the validity of this comparison assumes that a steady state condition has been reached and that the catalyst bed is not a source or a sink of carbon containing species.

For the (4.54 ± 0.02) μmol/mol methanol-in-air standard, the sum of the final methanol and formaldehyde amount of substance fractions agree with the initial methanol amount of substance fraction for both formaldehyde generator lengths. For the (88.8 ± 0.01) μmol/mol methanol standard, the sum of the final formaldehyde and methanol amount of substance fractions match the initial methanol amount of substance fraction for the generator with *l*_c_ = 11.4 cm and *l*_h_ = 7.6 cm. However, for the longer generator with *l*_c_ = 19 cm and *l*_h_ = 15.2 cm, the final formaldehyde and methanol amount of substance fractions are significantly lower than the initial methanol amount of substance fraction.

The FTS spectra shown in Figs. 5–7 indicate that CO_2_ is also present in both the methanol-in-air standards and in the generator outflow. Table 4 summarizes the initial and final CO_2_ amount of substance fractions for the generator using the (88.8 ±6 0.01) μmol/mol methanol standard. In each case, the CO_2_ amount of substance fraction in the generator exhaust is higher than the initial CO_2_ amount of substance fraction in the methanol standard. This suggests the catalyst converts some of the methanol to CO_2_. The net increase in the CO_2_ amount of substance fraction in the generator exhaust is also listed in Table 4. The amount of substance fraction of carbon products can be obtained by adding the net increase in CO_2_ to the sum of the final formaldehyde and methanol amount of substance fractions from Table 3. For the short generator, this final sum is equivalent to the initial methanol amount of substance fraction within the experimental uncertainties. However, for the longer generator, the final amount of substance fraction of the carbon products is less than the initial methanol amount of substance fraction. Therefore, either trace levels of other impurities containing carbon, such as those listed in Table 2, are also present in the generator exhaust or some carbon containing species remain in the generator.

The durability and lifetime of the catalyst was tested by repeated measurements over the course of several months as well as a 200 h continuous run of a 5 μmol/mol methanol-in-air standard over the catalyst. In both cases the catalyst conversion efficiency was consistent within our measurement uncertainty. This further suggests that a formaldehyde generator based on a molybdenum catalyst is a feasible method for producing low level standard amount of substance fractions of formaldehyde-in-air. Since the lifetime tests were limited, it is recommended that the catalytic conversion efficiency is periodically monitored until more lifetime data is acquired. Another improvement in the formaldehyde generator would be to incorporate an on-line real time monitor of the formaldehyde amount of substance fraction. Recent advances in compact diode and solid state laser systems are making this a more realistic possibility.

## 4. Summary

We have examined the feasibility of catalytically converting methanol-in-air mixtures to produce standard formaldehyde-in-air mixtures. A number of parameters for the catalytic conversion of methanol to formaldehyde were examined including: catalyst; catalyst bed and heater lengths; catalyst temperature; carrier gas; and the initial methanol amount of substance fraction. The highest observed conversion rate was (97 ± 4) % for the molybdenum catalyst. The conversion efficiency was found to be consistent over repeated cycles and over a long lifetime test, suggesting that a molybdenum catalyst is a viable candidate for a standard formaldehyde generator, particularly for low formaldehyde amount of substance fractions (< 15 μmol/mol). It is suggested that frequent calibrations should be made under the specific application conditions, since the catalyst lifetime and durability measurements have only been conducted under limited conditions. Furthermore, the strong dependence of the conversion efficiency on the initial methanol amount of substance fraction suggests that the generator must be calibrated for each specific amount of substance fraction required.

## Figures and Tables

**Table 1:** Catalysts tested for use in converting methanol to formaldehyde

Type	Particle diameter (mm)	Source
Chromium III oxide	3–6	Aldrich Chemical Company, Inc.
Copper	0.4–2	Aldrich Chemical Company, Inc.
Iron(III) oxide	0.3–0.6	Aldrich Chemical Company, Inc.
Molybdenum	0.8–1.6	Chemalloy Company, Inc.
Nickel	0.8–2	Envirotech
Silver	0.4–0.8	J&J Materials, Inc.

**Table 2:** Approximate Fourier-transform spectrometer detection limits of potential impurities in the formaldehyde generator outflow

Compound	4.54 μmol/mol methanol source (μmol/mol)	88.8 μmol/mol methanol source (μmol/mol)
Acetaldehyde	1	5
Formic acid	0.2	2
Methane	0.2	5

**Table 3:** Initial and final methanol amount of substance fractions determined by Fourier-transform infrared spectroscopy and final formaldehyde amount of substance fraction measured by tunable diode laser absorption spectroscopy. (The quoted uncertainties are expanded uncertainties with coverage factor *k* = 2 [14]

	Initial methanol amount of substance fractions		Post generator amount of substance fractions	
Generator length	Methanol (μmol/mol)	Methanol (μmol/mol)	Formaldehyde (μmol/mol)	Sum of methanol and formaldehyde (μmol/mol)
*l*_c_ = 11.4 cm, *l*_h_ = 7.6 cm	4.54 ± 0.02	0.5 ± 0.1	4.2 ± 0.15	4.7 ± 0.2
	88.8 ± 0.1	27.8 ± 0.5	59.8 ± 1.8	87.6 ± 1.9
*l*_c_ = 19 cm, *l*_h_ = 15.2 cm	4.54 ± 0.02	Not Determined	4.3 ± 0.15	4.3 ± 0.2
	88.8 ± 0.1	15.5 ± 0.5	67.3 ± 2.0	82.8 ± 2

**Table 4:** Initial and final carbon dioxide amount of substance fractions determined by Fourier-transform infrared spectroscopy. (The quoted uncertainties are expanded uncertainties with coverage factor *k* = 2 [14])

Generator length	Methanol sample(μmol/mol)	Initial CO_2_ (μmol/mol)	Post generator CO_2_ (μmol/mol)	Change in CO_2_ (μmol/mol)
*l*_c_ = 11.4 cm, *l*_h_ = 7.6 cm	88.8 ± 0.1	3.0 ± 0.1	4.4 ± 0.2	1.4 ± 0.2
*l*_c_ = 19 cm, *l*_h_ = 15.2 cm	88.8 ± 0.1	3.0 ± 0.1	5.2 ± 0.2	2.2 ± 0.2

## Footnotes

1 Certain commercial equipment, instruments, or materials are identified in this paper to foster understanding. Such identification does not imply recommendation or endorsement by the National Institute of Standards and Technology, nor does it imply that the materials or equipment identified are necessarily the best available for the purpose.

## 5. Appendix A. Measurement of Additional Products

The residual methanol was determined by directly comparing the integrated intensity of the absorbance spectra over a portion of the *v*8 band, 950 cm^−1^ to 1070 cm^−1^, with integrated intensities calculated from the NIST reference spectrum [16] of methanol absorption coefficients. The analysis was confined to the methanol CO stretch, since this band had the least interference from other species present. The band profile from the reference spectrum matched that of the sample spectrum except for formaldehyde features around 1075 cm^−1^.

The band profiles for CO_2_ and H_2_O in the sample spectra were distorted from those in the reference spectra [17]. This may be attributed to differences in the gas temperature and corresponding differences in the rotational and vibrational distributions of the CO_2_. The gas exiting the 350 °C catalyst bed may not completely equilibrate to 25 °C before the FTS measurement. The large rotational line spacing of CO_2_ and H_2_O makes this temperature effect more evident for these species compared to methanol which has unresolved bands. Estimates for the CO_2_ amount of substance fractions were obtained by integrating the absorbance spectra over the *v*_3_ band (2240 cm^−1^ to 2400 cm^−1^) and comparing the integrated intensities to that of the reference spectrum.
